# Techniques for Diagnosing Anastomotic Leaks Intraoperatively in Colorectal Surgeries: A Review

**DOI:** 10.7759/cureus.34168

**Published:** 2023-01-24

**Authors:** Sauvik Vardhan, Swati G Deshpande, Abhinesh Singh, Chava Aravind Kumar, Yuganshu T Bisen, Onkar R Dighe

**Affiliations:** 1 Department of General Surgery, Jawaharlal Nehru Medical College, Datta Meghe Institute of Higher Education and Research, Wardha, IND

**Keywords:** colorectal cancer, near-infrared fluorescence imaging, air leak test, intraoperative methods, anastomotic leak

## Abstract

Colorectal cancer is one of the most common surgically curable malignancies worldwide, having a good prognosis even with liver metastasis. This improved patient outcome is marred by anastomotic leaks (AL) in operated patients of colorectal cancer despite a microscopically margin-negative resection (R0). Various risk factors have been attributed to causing this. Preoperative non-modifiable factors are age, male sex, cancer cachexia, and neoadjuvant chemo-radiotherapy, and modifiable factors are comorbidities, peripheral vascular disease, anemia, and malnutrition. Intraoperative risk factors include intraoperative surgical duration, blood loss and transfusions, fluid management, oxygen saturation, surgical technique (stapled, handsewn, or compression devices), and approach (open, laparoscopic, or robotic). Postoperative factors like anemia, infection, fluid management, and blood transfusions also have an effect. With the advent of enhanced recovery after surgery (ERAS) protocols, many modifiable factors can be optimized to reduce the risk. Prevention is better than cure as the morbidity and mortality of AL are very high. There is still a need for an intraoperative technique to detect the viability of anastomotic ends to predict and prevent AL. Prompt diagnosis of an AL is the key. Many surgeons have proposed using methods like air leak tests, intraoperative endoscopy, Doppler ultrasound, and near-infrared fluorescence imaging to decrease the incidence of AL. All these methods can minimize AL, resulting in significant intraoperative alterations to surgical tactics. This narrative review covers the methods of assessing of integrity of anastomosis during the surgery, which can help prevent anastomotic leakage.

## Introduction and background

An anastomotic leak (AL) is described as a clinically significant fault at the anastomotic site that permits contact between the intraluminal and extraluminal compartments, as per the International Study Group of Rectal Cancer. Standard postoperative methods for its detection include digital rectal exploration, endoscopic examination, computed tomography radiographic evidence of contrast leakage through the suture gap, or the presence of perianastomotic hydro-aerial collection [1]. When bowel contents seep into the abdominal cavity, they can cause peritonitis leading to sepsis, shock, organ failure, or death. The incidence varies from 8% for the right-sided colon to 11% for rectal surgeries [2,3]. Reduced five-year survival rate and increased risk of local cancer recurrence in cases of malignancy are associated with anastomotic leaks [3]. Anastomotic leaks are estimated to increase healthcare expenses by 28.6 million dollars for every 1,000 patients receiving colorectal surgery [4]. Despite efforts to reduce its frequency, the incidence of the anastomotic leak has remained stable over the past several years resulting in a mortality of about 1.5% to 16.4% [5]. Apart from preoperative methods to optimize the patient and postoperative enhanced recovery after surgery (ERAS) protocols, this review aims to examine the various intraoperative methods in colorectal surgeries to detect and prevent anastomotic leakage. Table 1 shows the modifiable and non-modifiable risk factors for anastomotic leakage.

**Table 1:** Modifiable and non-modifiable risk factors ASA: American Society of Anesthesiologists; cm: centimeter

	Modifiable factors	Non-modifiable factors
Patient-related factors	Anemia	Male sex
	Malnutrition	Neo-adjuvant chemo-radiotherapy
	Serum protein level	
	Hypertension, hyperglycemia	
	Crohn's disease, coronary artery disease	
	Chronic steroid therapy	
	ASA scores of more than three	
Surgery-related factors	Antibiotic coverage	Distal site of the tumor
	Duration of surgery	Tumor size of more than 3 cm
	Iatrogenic injury	
	Contamination	
	Technique of anastomosis	

**Figure 1:**
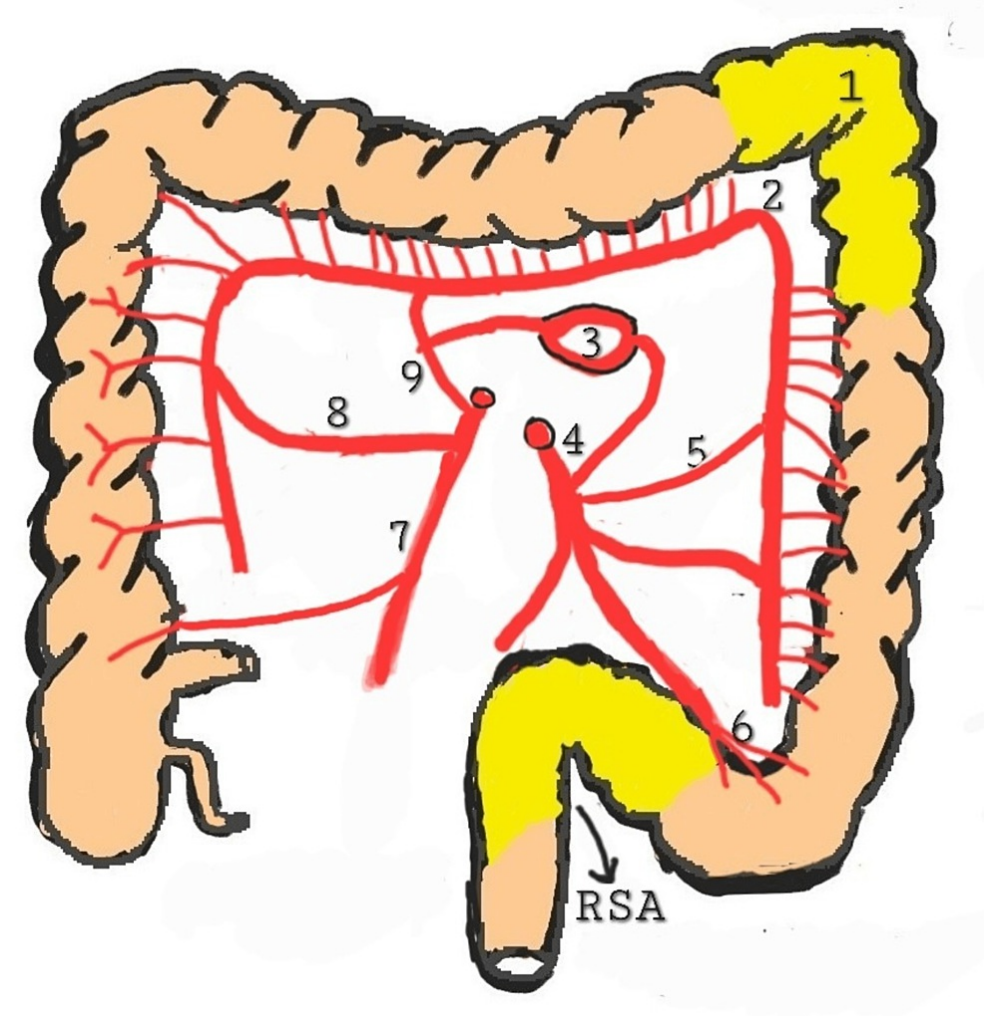
High-risk areas for anastomotic leak (1) Splenic flexure or Griffith's point; (2) absence of  Drummond's marginal arteries at Griffith's point; (3) arch of Riolan; (4) inferior mesenteric artery; (5) left colic artery; (6) sigmoidal arteries; (7) ileocolic artery; (8) right colic artery; (9) middle colic artery RSA: retrosigmoid area Source: Original image provided courtesy of the authors.

For the descending colon to receive an adequate blood supply, the middle colic artery, the marginal artery of Drummond, and consequently, the Riolan arch must all be in good health along with the left colic artery and inferior mesenteric artery. The splenic flexure (Griffith area), and the retrosigmoid, or Sudeck's point, are watershed areas. The risk of leakage from anastomosis in these areas is increased. Figure 1 shows the high-risk areas for the anastomotic leak. High ligation of the inferior mesenteric artery has been reported as a risk factor for anastomotic leak [6]. 

## Review

Methodology

**Figure 2:**
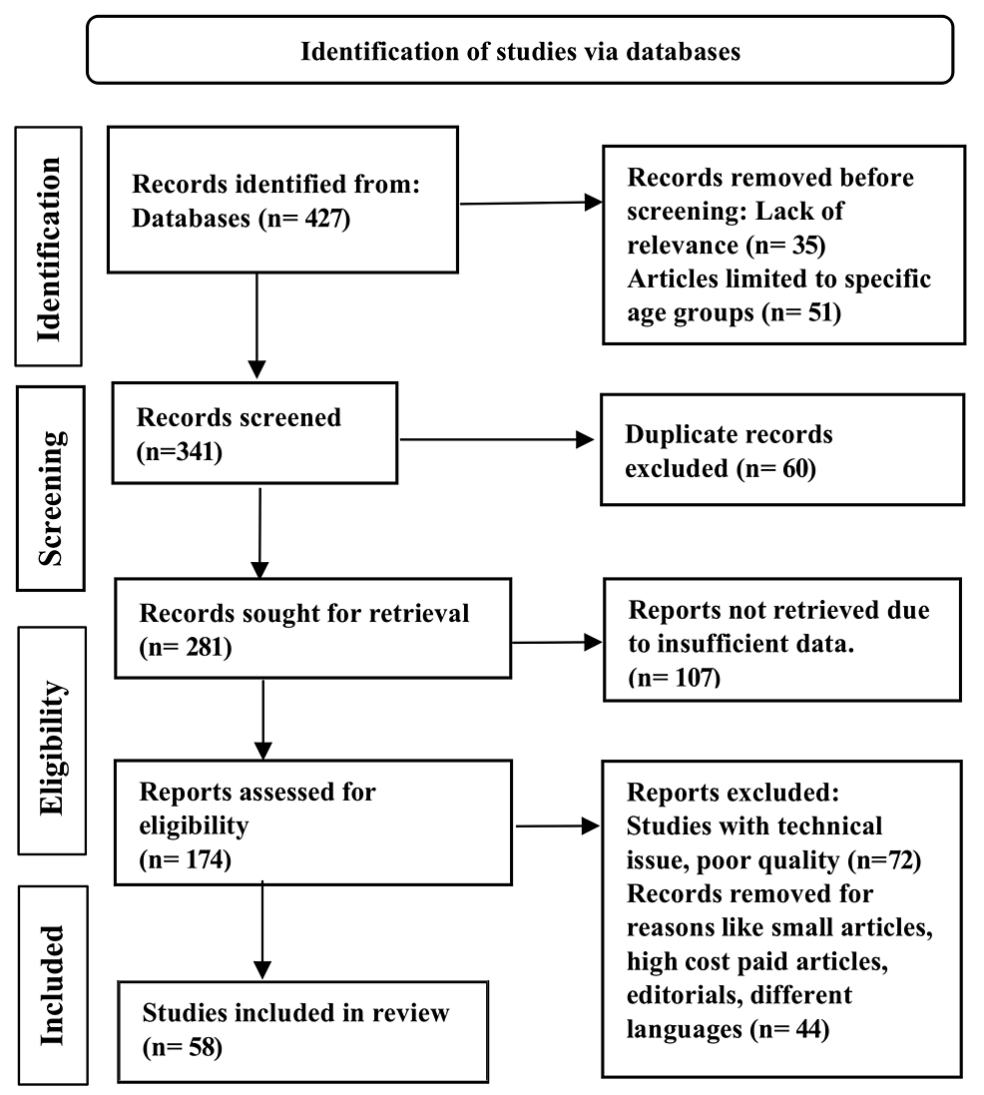
PRISMA model for the search strategy PRISMA: Preferred Reporting Items for Systemic Reviews and Meta-Analyses

Two separate reviewers, SV and SD, searched articles using a search of electronic databases such as PubMed, Google Scholar, and Researchgate. The search items were "anastomotic leak and intraoperative assessment methods", "anastomotic leak and intraoperative air leak test", "anastomotic leak and intraoperative colonoscopy", "anastomotic leak and intraoperative Doppler", and "anastomotic leak and near-infrared fluorescence imaging". Case reports were excluded and 58 articles were selected based on their effect on preventing anastomotic leaks using intraoperative methods. We also cross-checked the reference list of articles and included relevant articles. The methodology by the Preferred Reporting Items for Systemic Reviews and Meta-Analyses (PRISMA) method is shown in Figure 2 below.

Discussion

The incidence rate of anastomotic leak after colorectal surgery is between 1-19% [7]. Colorectal anastomotic leak and surgical site infection are the most common and important postoperative complications after colorectal surgery, causing pain and suffering to patients. In addition, these complications have been associated with negative economic impact, increased morbidity, extended postoperative hospital stay, readmission, sepsis, and death [8,9]. According to Fielding et al., it has been concluded that the surgeon is the most important individual factor influencing the integrity of anastomosis [10]. Methods used by the surgeon to control the surgical procedure and corrections are of utmost importance. The occurrence of an anastomotic leak also depends on the technique used for anastomosis. Handsewn and stapled anastomosis methods are still performed for anastomosis, reflecting the lack of agreement regarding the superiority of one over the other [11]. Robotic, laparoscopic, or open are the various approaches without any significant benefit of one over the other. The chances of anastomotic leakage are more when there is lower distal anastomosis. Numerous investigations have shown that anastomotic leak independently correlates with intra-operative transfusion [12,13] and intra-operative blood loss.

Intraoperative methods to diagnose anastomotic leaks

Several modes have been developed to check the integrity of colorectal anastomosis during surgery. These include crude subjective techniques like the color of the bowel edges, active bleeding from bowel edges used traditionally by surgeons to air leak tests, technically advanced intraoperative colonoscopy, intraoperative Doppler, near-infrared technology, and indocyanine green (ICG) angiography. These methods do not prevent anastomotic leaks but help predict the ones at increased risk, enabling the surgeon to take appropriate measures to correct them. Utilizing the bowel's color is one of the older techniques [14]. The gut is inflated at the anastomosis level to find a leakage from anastomosis, i.e., the air leak test, which can assist in identifying anastomotic leaks during surgery and facilitate their repair, decreasing the risk of postoperative anastomotic leaks. Intraoperative pulse oximetry is an inexpensive, convenient, rapid, and readily available method for assessing tissue viability. The disruption of anastomosis, in addition to determining the integrity of anastomosis, can be evaluated with the help of intraoperative colonoscopy [15]. Since then, several unequivocal and trustworthy techniques have been developed, most notably Doppler ultrasonography and near-infrared fluorescence technology. A key component of newer techniques is evaluating blood flowing to the anastomosis. Fluorescence perfusion angiography has lately demonstrated a broad range of therapeutic applications.

Pulse Oximetry

Pulse oximetry detects oxygen saturation at the end organ and represents an indirect measure of perfusion. Recent years have seen a lot of research into ways to use pulse oximetry for purposes other than just gauging arterial oxygen saturation from the fingertip or earlobe [16]. Experimental sensors based on reflectance pulse oximetry have been created for use in interior areas like the esophagus and colon. Some potentially relevant information concealed in these signals is starting to come to light through analysis of the photoplethysmography waveforms generated by these sensors. Various intra-operative oximeter designs are currently available for continuously monitoring the local tissue oxygen saturation (stO2). Some examples are the electrochemical sensor oximeter, reflectance electro-optical photoplethysmography probe oximeter, intrapartum pulse oximeter, and reflectance esophageal pulse oximeter. With a spectrum of benefits, their limitations include poor sensitivity, large probe size, interference from acidic stomach or bowel, and poor accuracy. A single-arm, prospective study by Salusjärvi et al. to measure the intra-operative colonic saturation with pulse oximetry from the wall of the colon between 2005 and 2011 found that there were 2.5% more anastomotic leaks occurring during operations when colonic stO2 was less than 90%. The risk for the same was found to be four times higher when stO2 values were less than 90% when logistic regression was performed [17]. The wireless pulse oximetry (WiPOX) device is a new wireless pulse oximeter. The onboard sensor-processor unit allows for non-invasive pulse oximetry as well as integration with current intraoperative monitoring. The contact pressure-sensing head provides accurate, high-quality measurements of the StO2 waveform despite the presence of physiological fluids. This tool can help surgeons choose an acceptable place for bowel anastomosis by focusing on well-perfused tissue with high healing potential. It can also help them discover anastomotic vascular compromise [18].

Air Leak Test

A regularly performed test to check the mechanical integrity of the anastomosis is the air leak test. It is performed by keeping the anastomosis in a pool of fluid (normal saline) and injecting a defined amount of air (50 cubic centimeters of air) per rectum. At the same time, the pressure inside the lumen is maintained by occluding the bowel proximal to the anastomosis. Air bubbles in the saline indicate a defect in the suture line and leakage through the anastomosis. If the bubbles were absent, the air testing was considered negative. The air testing procedure takes about three minutes [19]. Video 1 shows the air leak test. The limitation of the test is that patient anatomy varies, and it is difficult to ascertain the amount of air needed to raise the pressure across the anastomosis to the desired level from a biomechanical standpoint. In cases with positive air testing, the anastomotic defect was repaired by a single layer of extra mucosal sutures. After anastomotic reconstruction, air leak testing was repeated [19]. Lack of standardization in the air volume needs to be achieved in the set pressure, which should not exceed normal burst pressure to avoid false positive tests. It is important to stress that an undue increase in pressure across the anastomosis (either hand-sewn or stapled) may disrupt the anastomosis iatrogenically, hence weakening its integrity. According to a study by Schwab et al. in 2002, a newly formed colorectal anastomosis ruptures at a tension between 70-184 mmHg [20], even if a thorough analysis of the burst pressure is not yet available. It is still unclear whether performing ALT and immediately repairing ALT-positive cases is advantageous in averting colorectal anastomotic leak (CAL) despite being the most commonly performed test by the majority of colorectal surgeons. Due to the multifactorial etiology of CAL and varied ways of performing air leak tests by surgeons, air leak tests may only be less helpful in identifying colorectal anastomotic leakage caused by conditions apart from mechanical failure of anastomosis. According to a meta-analysis by Wu et al., of which 2395 out of 5283 patients were undergoing this test, no significant dissimilarity was there when compared to individuals who had not undergone ALT even though a lesser anastomotic leak rate was there in individuals with this test [21]. It helps to identify people with a greater risk of CAL, even if it cannot prevent them. The air leak test, which serves as the colorectal anastomosis quality control stage, urgently needs to be standardized in low-economy countries with poor resources.

**Video 1:** Negative air leak test Bowel after anastomosis is inflated with air in the saline pool but no air bubble was generated (as seen between nine seconds to 14 seconds in the video) implying a negative air leak test. Source: Video provided courtesy of the authors.

Colonoscopy Performed During Colorectal Surgery

Intraoperative colonoscopy (IOC) was first reported by Richter et al. in 1973 [22]. They located and identified impalpable lesions by laparotomy using intraoperative fiberoptic colonoscopy. Intraoperative colonoscopy includes the use of a colonoscope during laparoscopy or open surgery through the transected bowel end and inspecting proximal and distal colon to detect synchronous lesions, distal obstructive lesions, presence of inflammatory bowel disease, detect impalpable colonic lesions, especially in cases with the inability to complete preoperative colonoscopy, see the source of intestinal bleeding and to detect previous malignant polypectomy sites. This can also be done in cases of positive air leak tests. Intraoperative colonoscopy must be avoided when there is a clinically unstable patient. This also requires the surgeon to be well acquainted with colonoscopic techniques. Despite recent advancements in frequently used stapling devices, anastomosis-related issues, such as anastomotic hemorrhage, leakage, and colitis caused by insufficient blood flow or stricture, are still rarely observed according to research by Kawai et al. [23]. IOC, in particular, aids in detecting anastomotic bleeding and leak [24,25]. A 0.5-6.5% of stapled colorectal anastomosis instances have been reported to have postoperative bleeding [26-28]. Once the surgeon has detected anastomotic bleeding by intraoperative inspection of the staple line, hemostasis can be achieved with an additional suture or endoscopic clipping. According to Li et al., including a hemostatic suture in these situations decreased the risk of postoperative hemorrhage from 3.5% to 1%. They also found that intraoperative anastomotic bleeding occurred in 5.5% of patients who underwent IOC regularly. Nevertheless, because of the limited sample size, the difference was not statistically significant [15]. IOC and air leak testing can be combined to increase accuracy. Between 3-6% of patients, intraoperative air leakage from anastomoses has been found. If IOC hasn't been done regularly in these circumstances, postoperative anastomotic leaking should be anticipated. IOC may therefore be essential in determining the anastomotic integrity during surgery. It would be preferable to conduct a randomized study to determine whether regular IOC lowers postoperative anastomotic problems because IOC cannot totally prevent postoperative anastomotic bleeding or leaking. Combining mechanical techniques like IOC with modern digital techniques is desirable for better results in diagnosing the anastomotic leak. Limitations of IOC include that it is time-consuming, expensive, and needs technical expertise and further scientific research to effectively prevent anastomotic leaks [23].

Using Doppler During Colorectal Surgery

The intraoperative Doppler ultrasound of the marginal arteries is one intraoperative technique that is thought to predict the viability of the intestine in addition to clinical assessment [29]. According to earlier studies, the blood pressure in the marginal artery decreased by more than 30% when the left colic artery was blocked. Additionally, about 10% of patients experienced unstable blood flow to the left colon following inferior mesenteric artery (IMA) ligation, which could increase the rate of insufficient perfusion [30,31]. As it is simple to use and is a low-cost method, many surgeons suggested its use [32]. Vulnerability to the signals from large blood vessels nearby is considered a limitation of Doppler ultrasound. Doppler ultrasonography can impede local blood flow since it needs artery exposure, pulsatile blood flow, and tissue exposure, which further adds to its limitations. When the ends of the colon to be resected were examined with a Doppler ultrasonography, a 1% incidence of inadequate anastomosis was discovered in a study of a series of colorectal resections in roughly 200 patients [33]. But it was also observed that very little was added to the clinical judgment of anastomosis insufficiency. According to data revealed by Dyess et al. [34], Doppler ultrasonography readings frequently produced outcomes that were falsely positive as well as negative. Blood supply was examined with Doppler in at least two researches [35,36], but even though both found that blood flow was reduced following resection, it is unclear how much blood flow is required to prevent anastomotic leak [37,38]. The laser Doppler flowmetry was found to be superior to Doppler ultrasound in one study [39]. One extremely intriguing potential non-invasive tool for analysis of the amount of blood flowing in the intestine is the laser Doppler flowmetry. According to a study [40], different amounts of tissue blood flow were discovered using laser Doppler velocimetry to be connected to the wound healing process at the anastomosis site. Anastomosis is regarded as healthy and dehiscence is uncommon when the laser Doppler velocimetry value is one. This represents roughly 30% of tissue blood flow of the wall of the colon which is intact. Laser Doppler flowmetry was employed in the study cited by Hallböök et al. [41] to assess transmural colonic blood flow before creating a plain or pouch anastomosis during surgery. The vascular supply was measured at two places before dissecting the intestine: one, 8 cm distant, and second, close to the targeted intestinal end. A second recording was taken at the exact locations after dissection and, where feasible, pouch generation but before the anastomosis was finished. In the end-to-end anastomosis population, blood flow levels at the location designated for the anastomosis were significantly decreased after colon dissection. Blood supply levels at the anastomotic site were comparable in the pouch population after intestinal dissection and pouch construction (side-to-end anastomosis). It is suggestive from results that, at the pouch anastomosis site, unaffected blood supply may be helpful for recovery of anastomosis. Additionally, when it comes to the small intestine partner of an anastomosis, such as the ileocolic anastomosis following right hemicolectomy or the creation of a small bowel J-pouch, the indocyanine green quantitative flow technique now enables more accurate blood flow measurement. However, the research by Johansson and colleagues shows that laser Doppler may still be used for direction [42].

Indocyanine Green and Near-infrared Fluorescence Technology

The most used fluorescent probe is indocyanine green (ICG), which has been authorized for clinical use by the Food and Drug Administration (FDA) since 1959 [43]. Fluorescence can be seen immediately on the surgical field, on a screen, during minimally invasive operations, and during open surgical procedures. On the best time to conduct the perfusion assessment, there is disagreement. Using near-infrared fluorescence imaging (NIRF), surgeons may see the bowel's micro-perfusion in real time [44]. This method has been demonstrated to be workable in determining the vascularization of the colon that will be anastomosed when perioperative ICG injection is used. Laparoscopic anastomotic bowel surgery appears to be more successful and safer when fluorescence angiography is used in conjunction with intraoperative ICG injection [44,45]. However, no impartial quantification of the signal was carried out in investigations supporting the reduced prevalence of AL [46-51]. To make the approach a trustworthy instrument for correctly and consistently assessing intestinal perfusion at the anastomotic location in real-time, such quantification is necessary. Potentially given by the intravenous route, ICG is a kind of iodine dye. ICG, which has a half-life of three to five minutes, is broken down by the liver into bile in 15-20 minutes and has no recognized metabolites [52]. The use of intravenous ICG is typically regarded as being fairly safe, and vasovagal or allergic symptoms such as anaphylactic shock, hypotension, tachycardia, dyspnea, or urticaria are extremely infrequent. ICG is the best agent for obtaining high-quality pictures of the lymphatic and circulatory systems due to these characteristics [53-55]. The ICG gets excited between 750 nanometers to 800 nanometers. Fluorescence is seen around 830 nanometers [56]. During surgery, when NIRF is used, a colorectal surgeon can see the vascularity of the bowel. Lymph nodes draining the cancer site along with ureters can also be assessed. Augmented reality (AR) is increasing in operation theatres so that surgeons can be assisted. AR is created when a real-time image that is viewed by a device is superimposed with digital information. It allows us to enlarge our insight into reality. The ability to absorb a shorter wavelength while discharging a longer wavelength is fluorescence. Fluorochrome discharging in the near-infrared spectrum is given by the intravenous route or directly into the tissues during NIRF imaging. Like a laparoscope, the AR acquisition system works similarly but employs two cameras simultaneously. For real-world images, a white light camera, and for fluorescence images discharged by the dye, there is a second infrared camera. On a black background, the fluorochrome rays are seen on fluorescent images as white pixels. By superimposing the real-world image on top of recolored near-infrared (NIR) image, the AR image is created. Just like light, radiation also pierces biological tissue. The energy gets gradually consumed on its way to the depth of tissue and gets absorbed. The living tissue absorbs and scatters the shorter wavelengths, and NIR light pierces living tissues (skin and blood) more effectively than visible light. Due to this, the tissues have a "transparency" effect, allowing the fluorescent images to be acquired through many millimeters of depth. As per Jafari et al. [48], there was a reduction in anastomotic leakage from 18% to 6% following anterior resection with the use of fluorescence perfusion angiography intraoperatively. NIRF imaging can fulfill three main purposes: (1) determining the number of ischemic segments during acute intestinal ischemia or estimating intestinal vessels supplying blood to identify regions of inadequate perfusion and avoid AL; second, avoiding recurrence and modifying additional adjuvant therapy as it is important to see lymphatic drainage, sentinel lymph nodes (SLNs), and lymphatic and peritoneal metastases, especially in oncological situations; and third, allowing the recognition of ureters to lower the danger of iatrogenic ureteral injuries, especially during laparoscopic surgery [57].

**Figure 3:**
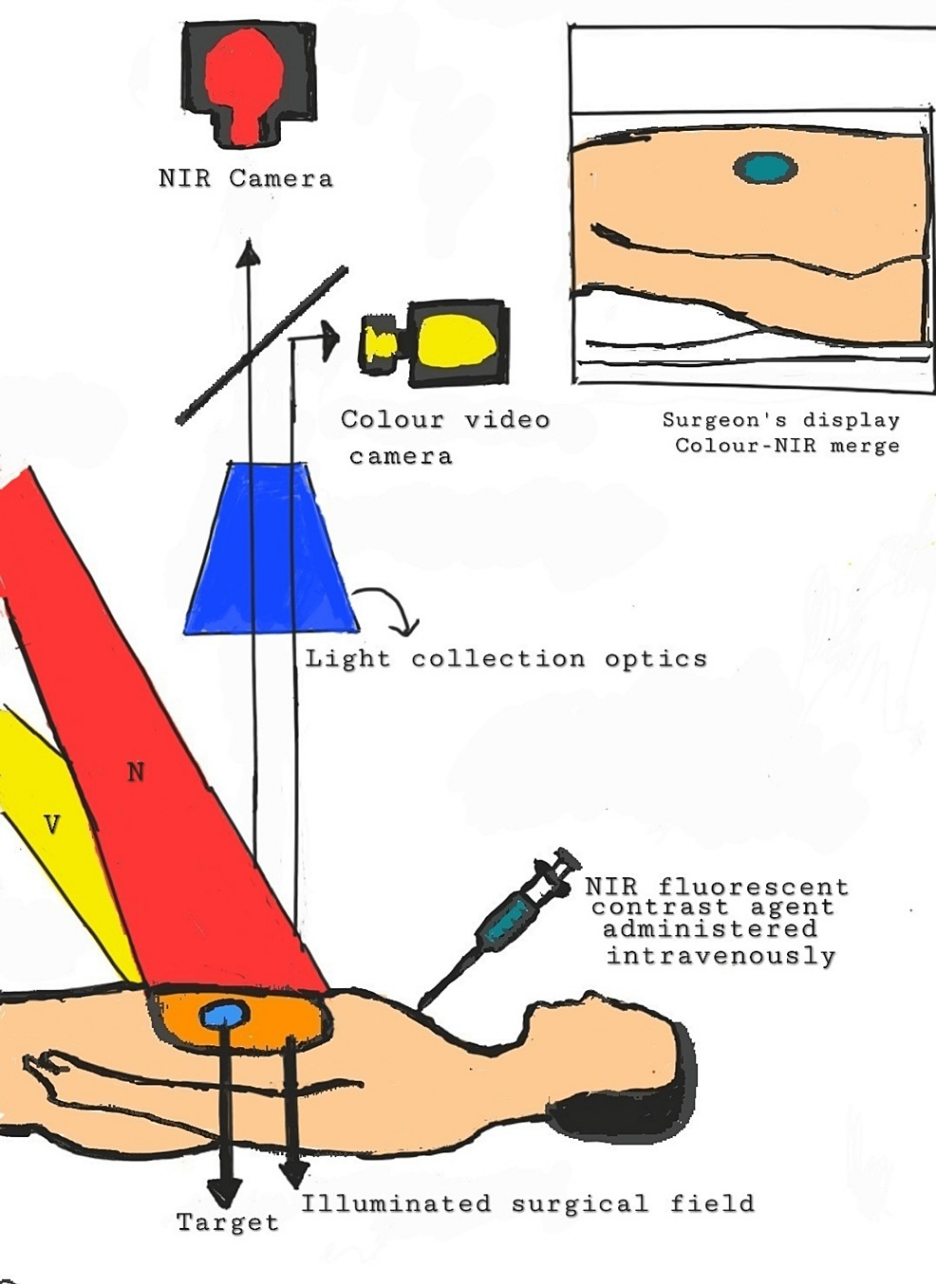
Visualization process in NIRF imaging NIRF: near-infrared fluorescence imaging; NIR: near infra-red; V: Visible light; N: NIR light source Source: Original image provided courtesy of the authors.

The fluorescence perfusion angiography changed bowel division strategy due to insufficient perfusion in around 6% of patients of a total of 504 patients, with no subsequent anastomotic leakage, as per a prospective cohort study. According to the PILLAR II trial, 8% of the 139 patients who underwent anterior resection without subsequent anastomotic leak (AL) had their surgical plan modified [46]. A recent systematic review and meta-analysis of 1302 individuals confirmed these findings. It was reported that the rate of AL in colorectal cancer patients undergoing surgery was reduced by fluorescence perfusion angiography. Following that, a meta-analysis of 10 trials from 2010 to 2017 by Van den Bos et al. found that 10.8% of patients had a change in surgical strategy (894 patients) [44]. In a case-matched retrospective research, Kudszus et al. [50] found that utilizing fluorescence angiography reduced the probability of surgical revision by 60%. Jafari et al. performed robotic-assisted laparoscopic rectal surgery to corroborate these findings [48]. In the majority of the investigations, the anastomosis was repeated if the tissue was poorly perfused or the resection was extended to well-perfused tissues. Figure 3 shows the visualization process of anastomosis in colorectal surgery. Although certain studies have demonstrated various benefits of using NIRF imaging intraoperatively, there are limitations like, use of repeated ICG injections have not been studied or known to have any effects [58]. The use of ICG and NIRF is dependent on equipment and is not available to peripheral hospitals due to associated costs. Another limitation is that there is yet no precise analytical method to evaluate the signal strength objectively and that the surgeon's opinion is still required for picture interpretation. Despite the limitations, ICG-enhanced fluorescence imaging appears to be a reliable, safe, and affordable method for determining colonic and anastomotic perfusion. Table 2 shows the comparison between some of the methods that can be done intraoperatively.

**Table 2:** Comparison between features of various intraoperative techniques for checking anastomotic leak +: Present; -: Absent

Intraoperative technique	Can be used in laparoscopic surgery	Ease of usage	Accuracy	Objective	Reproducible	Cost-effective
Colour of the bowel	+	+	low	-	+/-	+
Marginal blood vessel	+	+	low	-	+/-	+
Pulse oximetry	+	+	low	+	+	+/-
Colonoscopy	+	+/-	high	+/-	+	+/-
Doppler ultrasonography	+	+	low/high	+/-	+/-	+
Near-infrared fluorescence technology	+	+ +	high	+	+	++

## Conclusions

Anastomotic leak (AL) is a very threatening complication in colorectal surgeries. Its occurrence has been relatively stable over the last few years. Various methods have been suggested by surgeons to diagnose and eliminate the anastomotic leak intraoperatively. It is crucial to combine older techniques with newer techniques, such as the air leak test, to evaluate the anastomosis integrity and the absence of AL. Air leak tests can predict the risk of an anastomotic leak by acting as a quality check method for anastomosis. It should be promoted in countries with poor resources and economies. Using endoscopy in between the procedure is also beneficial in preventing AL. Nevertheless, much expertise is required for this procedure during the surgery and additional operative time may get consumed while performing the same. Using ultrasound, we can measure the suture line blood flow in colonic anastomosis made with a stapler, manual suturing, or a combination. The occurrence of AL can be further decreased by near-infrared fluorescence imaging (NIRF). There is a need to implement NIRF imaging in colorectal surgeries as it can be used for three crucial indications such as assessing the supply of blood, application of oncology, and highlighting anatomical structures like ureters. As NIRF technology is cost-effective, easy to use, and can be used in laparoscopic surgeries with certain other benefits, its use should be promoted during colorectal surgeries.
